# Association of *CTLA-4* tagging polymorphisms and haplotypes with hepatocellular carcinoma risk: A case-control study

**DOI:** 10.1097/MD.0000000000016266

**Published:** 2019-07-19

**Authors:** Jing Yang, Jiaochun Liu, Yu Chen, Weifeng Tang, Chao Liu, Yuling Sun, Jianping Chen

**Affiliations:** a Department of Gastroenterology, The Third Affiliated Hospital of Soochow University, Changzhou, Jiangsu Province; b Department of Gastroenterology, People's Liberation Army 92nd Hospital, Nanping; c Cancer Bio-immunotherapy Center; d Department of Medical Oncology, Fujian Cancer Hospital & Fujian Medical University Cancer Hospital; e Fujian Provincial Key Laboratory of Translational Cancer Medicine, Fuzhou, Fujian Province; f Department of Cardiothoracic Surgery, Affiliated People's Hospital of Jiangsu University, Zhenjiang, Jiangsu Province; g Department of Hepatobiliary and Pancreatic Surgery, The First Affiliated Hospital of Zhengzhou University; h Institute of Hepatobiliary and Pancreatic Diseases, Zhengzhou University, Zhengzhou, Henan Province, China.

**Keywords:** CTLA-4, haplotype, hepatocellular carcinoma, polymorphism, risk

## Abstract

It has been proposed that cytotoxic T-lymphocyte antigen 4 (CTLA-4) may attenuate the T-cell activation threshold, thereby decreasing the antitumor response and conferring susceptibility to hepatocellular carcinoma (HCC).

In the present study, we selected *CTLA-4* tagging single nucleotide polymorphisms (SNPs) and explored the relationship between these polymorphisms and susceptibility to HCC. A hospital-based case-control study, comprising 584 cases with HCC and 923 controls, was performed in an eastern Chinese Han population. *CTLA-4* SNPs were genotyped using a custom-by-design 48-Plex SNPscan Kit.

We found that the *CTLA-4* rs3087243 G>A polymorphism might be associated with increased risk of HCC (GA vs GG: adjusted odds ratio [OR], 1.38; 95% confidence interval [CI], 1.04–1.85; *P* = .028 and AA/GA vs GG: adjusted OR, 1.43; 95% CI, 1.08–1.89; *P* = .012). After using Bonferroni correction, this association remained (*P* = .012 for the AA/GA vs GG genetic model). In addition, the power value was 0.904 in the AA/GA versus GG genetic model. Haplotype analysis showed that *CTLA4* C_rs16840252_A_rs231775_A_rs3087243_T_rs733618,_ C_rs16840252_G_rs231775_A_rs3087243_T_rs733618_, and other haplotypes might increase the risk of HCC risk (*P* = .018, <.001, and .017, respectively). However, we found that *CTLA4* T_rs16840252_A _rs231775_G_rs3087243_T_rs733618_ decreased the risk of HCC (*P* = .020).

Our results suggest that the *CTLA-4* rs3087243 G>A polymorphism increases susceptibility to HCC in an eastern Chinese Han population. *CTLA-4* haplotypes may influence the development of HCC. In the future, a population-based fine-mapping study with functional assessment should be performed to further determine these potential correlations.

## 1 Introduction

In 2012, an estimated 782,500 new liver cancer cases were diagnosed worldwide, accounting for approximately 5.55% of all cancer patients.^[1]^ In addition, there were 745,500 liver cancer-related deaths.^[1]^ China has a high rate of hepatocellular carcinoma (HCC) accounting for more than 50% of liver cancer cases and deaths.^[2]^ Most primary liver cancers are HCC. The high incidence of HCC in Asia and sub-Saharan Africa mainly reflects the elevated levels of hepatitis B virus (HBV) infection. The high HBV infection rate is thought to be associated with the development of HCC. However, chronic HBV infection does not account for all of the etiology of HCC. Recently, investigations have focused on the association between heredity factors and the risk of HCC. Thus, the exploration of potential genetic factors might be beneficial to the diagnosis and prevention of HCC.

Chronic virus infection and inflammation might contribute to the development of HCC. Cytotoxic T-lymphocyte antigen 4 (CTLA4) is considered a vital negative regulator of immune responses.^[3]^ CTLA4, a member of the immunoglobulin superfamily (IgSF), is expressed on activated T cells and inhibits their function. CTLA4 is homologous to CD28, both of which bind to CD80 and CD86. CTLA-4 has greater affinity and avidity than CD28 when binding with CD80 and CD86. CTLA4 inhibits the function of T cells^[4]^; however, CD28 transmits a stimulatory signal to T cells. In addition, CTLA4 is also found on the surface of regulatory T cells and leads to its inhibitory function. CTLA-4 binds with B7 to represses T cells at the G1 phase and decreases the expression of interleukin-2 (IL-2) and the IL-2 receptor.^[5]^ CTLA-4 can also induce Fas cell surface death receptor-independent apoptosis of activated T lymphocytes, and then further restrain T cells.^[6]^ It has been proposed that, during the development of malignancy, CTLA-4 may attenuate the T-cell activation threshold, thereby decreasing the antitumor response and conferring susceptibility to cancer.

Recently, case-control studies have focused on the association of the *CTLA-4* single-nucleotide polymorphism (SNP) rs231775 G>A with the risk of HCC.^[7–9]^ However, Hu et al found that *CTLA-4* rs231775 G>A did not confer susceptibility to HCC.^[8]^ Thus, because of the limited numbers of case-control study, the relationship between *CTLA-4* rs231775 G>A with HCC susceptibility is not well established. In addition, other important SNPs in the *CTLA-4* gene may be associated with the risk of HCC, whose potential correlations are unknown. Therefore, in the present study, we selected *CTLA-4* tagging SNPs (rs733618 T>C, rs231775 G>A, rs3087243 G>A, and rs16840252 C>T) and explored the relationship between these polymorphisms and susceptibility to HCC.

## 2 Materials and methods

### 2.1 Study population and patient selection

A hospital-based case-control study comprising 584 patients with HCC (age range, 20–83 years) and 923 controls (age range, 21–80 years) was performed between January 2002 and December 2016 in an eastern Chinese Han population. All the HCC cases and controls were recruited from Fuzong Clinical Medical College and Union Clinical Medical College of Fujian Medical University. The major enrollment criteria for the patients with HCC were:

(1) Pathologically diagnosed HCC;
(2) living in eastern China for more than 10 years, and
(3) not treated with chemoradiotherapy.

The major exclusion criteria were:

(1) Patients with HCC who received prior chemoradiotherapy;
(2) cases of HCC with autoimmune disease;
(3) cases only diagnosed using ultrasound; and
(4) patients with a history of another malignancy.

In total, 923 healthy subjects who attended the hospital for a routine physical examination were enrolled as noncancer controls. The eligibility criteria for the controls were:

(1) No chronic liver disease;
(2) no autoimmune disease; and
(3) no history of malignancy.

All subjects were from the Chinese Han population and were unrelated. All participants were informed about the aim of study in an interview and provided written consent. Information on the demographic factors, risk factors, and clinical characteristics of the HCC cases and controls was collected using a questionnaire. The study protocol was approved by the Ethical Committee of Fujian Medical University, and conformed to the principles of the Helsinki Declaration. HCC cases and controls were full-matched by age and sex (Table 1). The criteria for “ever smokers” and “ever drinkers” were presented in our previous study.^[10]^

**Table 1:**
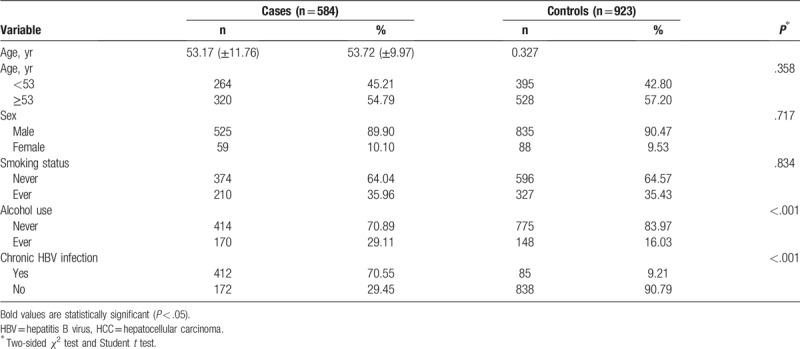
Distribution of selected demographic variables and risk factors in HCC cases and controls.

### 2.2 Selection of CTLA-4 tagging SNPs

Using the Genome Variation Server data (http://gvs.gs.washington.edu/GVS147/), *CTLA-4* tagging SNPs were selected. The major criterion were:

(1) the data came from a Chinese Han in Beijing cohort;
(2) a *P* value for the Hardy–Weinberg equilibrium (HWE) of no less than .05;
(3) the minor allele frequency was more than 0.05; and
(4) a pairwise linkage disequilibrium *r*^*2*^ threshold of 0.8 between SNPs (*r*^2^ > 0.8); and
(5) a call rate ≥95%.^[10,11]^

Finally, 4 *CTLA-4* tagging SNPs (rs733618 T>C, rs231775 G>A, rs3087243 G>A, and rs16840252 C>T) were selected as eligible for the study. Table 2 presents important information for these polymorphisms.

**Table 2:**
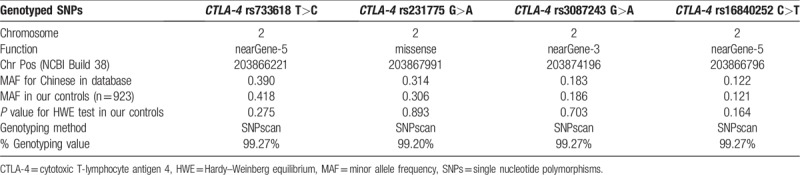
Primary information for *CTLA-4* polymorphisms (rs3087243 G>A, rs16840252 C>T, rs733618 T>C, and rs231775 G>A).

### 2.3 DNA extraction and genotyping

Genomic DNA was carefully extracted from 2 ml of EDTA-anticoagulated peripheral blood using a Promega DNA kit (Promega, Madison, WI) according to the manufacturer's instructions (www.promega.com/protocols/). The *CTLA-4* tagging SNPs were genotyped using a custom-by-design 48-Plex SNPscan Kit (Genesky Biotechnologies Inc, Shanghai, China) as described in previous studies.^[12–14]^ Sixty (4%) DNA samples were randomly selected and genotyped again by another laboratory technician, and the reproducibility was 100%.

### 2.4 Statistical analysis

The differences in demographic or risk factors were calculated using Student *t* test or Pearson 2-sided *χ*^*2*^ test when appropriate. Consistency of the genotype frequencies with the HWE for each *CTLA-4* tagging SNP among the controls was measured using an online Pearson 2-sided *χ*^*2*^ test (http://ihg.gsf.de/cgi-bin/hw/hwa1.pl).^[15]^ Pearson *χ*^*2*^ or Fisher's exact test were also used to assess the differences in the genotype frequencies between 2 groups. The distribution of *CTLA-4* tagging SNPs genotype frequencies in the different groups was compared. To adjust for confounding factors (eg, the status of chronic HBV infection, age, sex, smoking, and alcohol use), logistic regression analysis was also performed. The odds ratios (ORs) were calculated to assess the associations and for all adjusted ORs, the 95% confidence intervals (95% CI) were calculated. SAS software (version 9.4, SAS Inc. Cary, NC) was used for the statistical analysis. Significance was assumed for *P* < .05 (2-tailed). In this study, a Bonferroni correction test was applied to conduct multiple testing.^[16,17]^ We assessed the power of the present study (*α* = 0.05) using the PS Calculator (http://biostat.mc.vanderbilt.edu/twiki/bin/view/Main/PowerSampleSize). We used an expectation-maximization algorithm (SHESIS program [Bio-X Inc, Shanghai, China, http://analysis.bio-x.cn/myAnalysis.php])^[18]^ to conduct the haplotype analysis.

## 3 Results

### 3.1 Demographic characteristics

The demographic characteristics of the participants are summarized in Table 1. A total of 584 patients with HCC (male:female = 525:59; mean age: 53.17 ± 11.76 years) and 923 control subjects (male:female = 835:88; mean age: 53.72 ± 9.97 years) participated in this case-control study. The status of chronic HBV infection, smoking, and alcohol use of both groups are presented in Table 1. There were no significant differences in the distribution of sex, age, and smoking between the patients with HCC and the controls (*P* = .717, .327, and .834, respectively). The rate of chronic HBV infection was higher for patients with HCC than for the controls (70.55% vs 9.21%; *P* < .001). In addition, alcohol use was significantly higher for the patients with HCC than for the controls (29.11% vs 16.03%; *P* < .001, respectively). As shown in Table 2, the genotyping success rates ranged from 99.20% to 99.27%. The distribution of the *CTLA-4* rs733618 T>C, rs231775 G>A, rs3087243 G>A, and rs16840252 C>T genotypes among the controls conformed to the HWE.

### 3.2 Association of CTLA-4 rs733618 T>C, rs231775 G>A, rs3087243 G>A, and rs16840252 C>T polymorphisms with susceptibility to HCC

The *CTLA-4* rs733618 T>C, rs231775 G>A, rs3087243 G>A, and rs16840252 C>T genotypes are listed in Table 3.

**Table 3:**
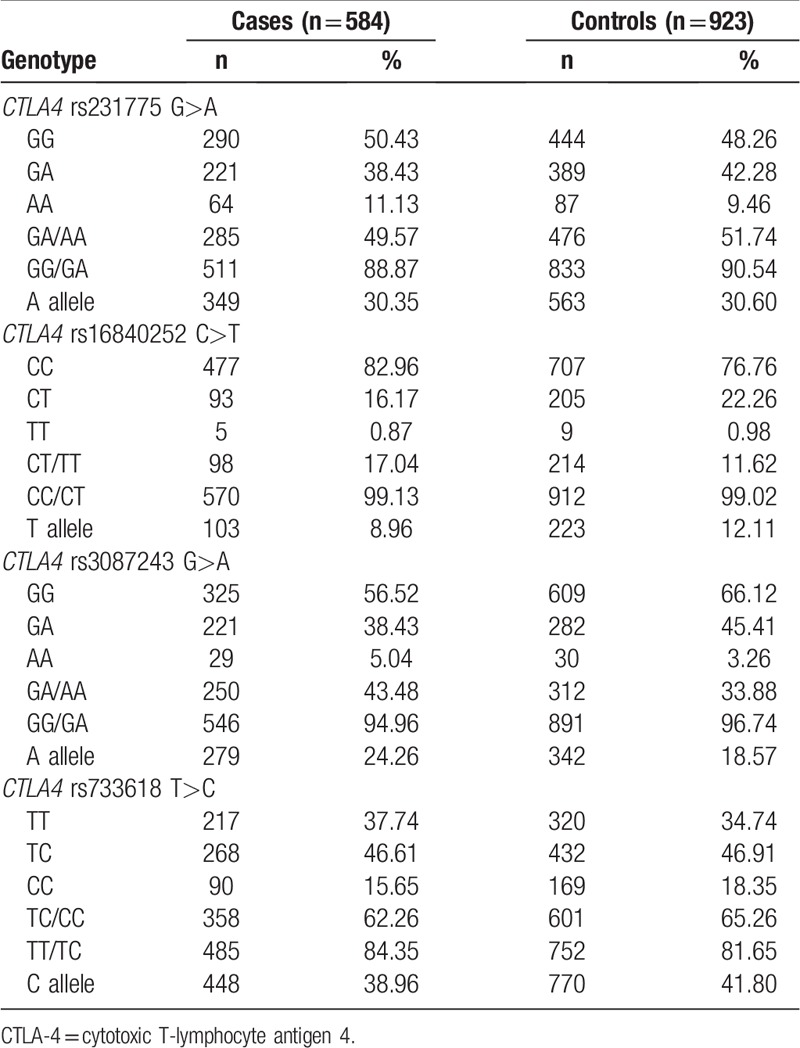
Genotypes of *CTLA-4* rs3087243 G>A, rs16840252 C>T, rs733618 T>C, and rs231775 G>A polymorphisms.

The frequencies of the *CTLA-4* rs231775 GG, GA, and AA genotypes were 50.43%, 38.43% and 11.13% in the HCC cases and 48.26%, 42.28%, and 9.46% in the controls, respectively. After adjusting for chronic HBV infection status, age, sex, smoking, and drinking, we found that the *CTLA-4* rs231775 G>A polymorphism might decrease the risk of HCC (GA vs GG: adjusted OR = 0.74, 95% CI: 0.55–0.99, *P* = .042; AA vs GG: adjusted OR = 1.16, 95% CI: 0.73–1.84, *P* = .519; GA/AA vs GG: adjusted OR = 0.83, 95% CI: 0.63–1.09, *P* = .168 and AA vs GG/GA: adjusted OR = 1.34, 95% CI: 0.86–2.08, *P* = .193; Table 4).

**Table 4:**
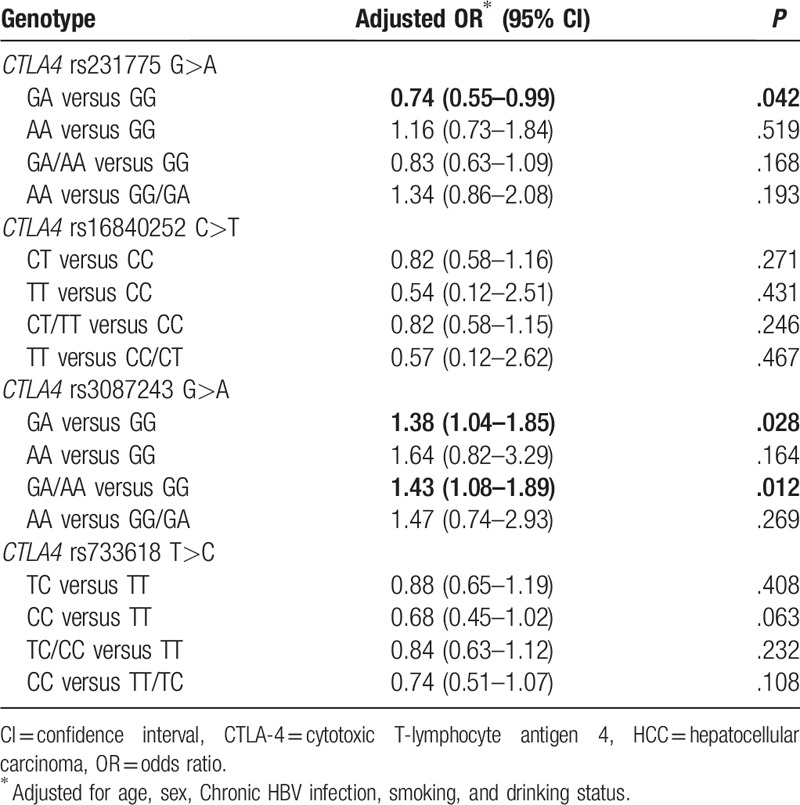
Logistic regression analyses of associations between *CTLA-4* rs3087243 G>A, rs16840252 C>T, rs733618 T>C, and rs231775 G>A polymorphisms and the risk of HCC.

The frequencies of the *CTLA-4* rs3087243 GG, GA, and AA genotypes were 56.52%, 38.43%, and 5.04% in the HCC cases and 66.12%, 45.41%, and 3.26% in the controls, respectively. After adjusting for chronic HBV infection status, age, sex, smoking, and drinking, an association between the *CTLA-4* rs3087243 G>A polymorphism and increased risk of HCC was found (GA vs GG: adjusted OR, 1.38; 95% CI, 1.04–1.85; *P* = .028 and AA/GA vs GG: adjusted OR, 1.43; 95% CI, 1.08–1.89; *P* = .012; Table 4). For the *CTLA-4* rs3087243 G>A polymorphism, the power value was 0.904 in the AA/GA vs GG genetic model. After using Bonferroni correction for multiple tests, we found that the *CTLA-4* rs3087243 G>A polymorphism was still associated with an increased risk of HCC (*P* = .012 for the AA/GA vs GG genetic model). Other results of the Bonferroni correction test are not shown.

By contrast, we found that the *CTLA-4* rs16840252 C>T and rs733618 T>C polymorphisms were not associated with susceptibility to HCC (Table 4).

### 3.3 SNP haplotypes

Results of haplotype analysis suggested that *CTLA4* C_rs16840252_A_rs231775_A_rs3087243_T_rs733618,_ C_rs16840252_G_rs231775_A_rs3087243_T_rs733618_, and other haplotypes significantly increased the risk of HCC (*P* = .018, <.001, and .017, respectively, Table 5). However, we also found that *CTLA4* T_rs16840252_A_rs231775_G_rs3087243_T_rs733618_ decreased the risk of HCC (*P* = .020, Table 5).

**Table 5:**
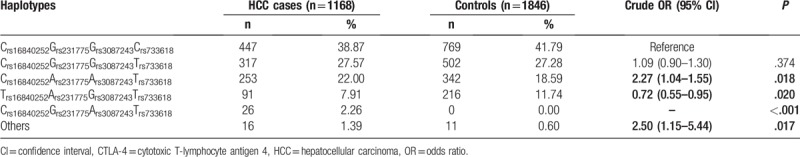
*CTLA4* haplotype frequencies (%) in cases and controls and risk of HCC.

## 4 Discussion

CTLA-4, an IgSF receptor, plays an important role in immune regulation by feeding a negative signal to T cells once an immune response has been initiated and completed.^[19]^ In this case-control study, the role of *CTLA-4* tagging polymorphisms (rs733618 T>C, rs231775 G>A, rs3087243 G>A, and rs16840252 C>T) in HCC susceptibility was assessed. We found that the *CTLA-4* rs3087243 G>A polymorphism was associated with an increased risk of HCC, and this association remained after Bonferroni correction.

Polymorphism rs3087243 G>A is located in the 3′-untranslated region of the *CTLA-4* gene. It has also been named as CTLA4 60G/A. A meta-analysis reported that the *CTLA-4* rs3087243 G>A polymorphism increased susceptibility to skin cancer.^[20]^ However, a pooled-analysis also suggested that *CTLA-4* rs3087243 G>A might decrease the risk of breast cancer.^[21]^ Thus, these previous results were conflicting and ambiguous. Considering the common variants conferring a low penetrance susceptibility to the development of cancer, we enrolled 1507 participants and conducted a case-control study to obtain a more precise evaluation. In this study, we found that the *CTLA-4* rs3087243 A allele was associated with an increased risk of HCC. To further confirm this potential association, Bonferroni correction was performed, after which this association remained valid. In addition, the power of this case-control study (*α* = 0.05) was evaluated using an internet-based Power and Sample Size Calculator. The power value was 0.904 in the AA/GA versus GG genetic model, which suggested a potential association. Recently, a functional study found lower expression of the soluble (s)-CTLA4 isoform compared with the membrane-bound CTLA4 in lymphocytes from patients with inflammatory bowel disease with the *CTLA-4* rs3087243 GG genotype compared with that of the AA genotype.^[22]^ These results indicated that the *CTLA-4* rs3087243 G→A variant might increase the expression of CTLA4, thereby elevating the T-cell activation threshold, leading to a weakened antitumor response and conferring a risk of HCC. However, in the present study, because of the moderate sample size, these primary findings should be explained with very caution. In the future, further investigations should be performed to verify these correlations.

As summarized in Table 5, the haplotype analysis indicated that the frequency of *CTLA4* C_rs16840252_A_rs231775_A_rs3087243_T_rs733618,_ C_rs16840252_G_rs231775_A_rs3087243_T_rs733618_, and other haplotypes significantly increased the risk of HCC. However, we also found that *CTLA4* T_rs16840252_A_rs231775_G_rs3087243_T_rs733618_ decreased the risk of HCC. We first explored the potential relationship of these *CTLA-4* haplotypes with the development of HCC. A previous study highlighted that the *CTLA4* C_rs16840252_G_rs231775_A_rs3087243_T_rs733618_ haplotype might increase susceptibility to gastric cardia adenocarcinoma,^[15]^ which was similar to the results of the present study.

The present study has some merits. First, we selected tagging SNPs to explore the potential relationship of *CTLA-4* variants with HCC susceptibility. Second, to the best of our knowledge, this was the first case-control study to explore the association of *CTLA-4* rs733618 T>C, rs3087243 G>A, and rs16840252 C>T polymorphisms with HCC. Finally, the distribution of *CTLA-4* rs733618 T>C, rs231775 G>A, rs3087243 G>A, and rs16840252 C>T genotypes among controls conformed to HWE, which suggested our findings were less prone to bias.

Some limitations of the study should be acknowledged. First, our case-control study only included eastern Chinese Han population; thus, the findings are only applicable to this ethnicity. Second, the sample size of the present study was moderate. Further large-scale and well-designed studies taking into account detailed environmental factors are needed to confirm this relationship in various populations. Third, we lacked sufficient information on the prognosis of HCC among the patients; therefore, we could not further analyze the potential role of *CTLA-4* variants in HCC survival. Finally, although we took several risk factors into consideration, such as chronic HBV infection status, sex, age, drinking, and smoking status, many other environmental factors and lifestyle parameters that might be related to the risk of HCC, were not assessed.

In conclusion, our results highlighted that the *CTLA-4* rs3087243 G>A polymorphism was associated with susceptibility to HCC in an eastern Chinese Han population. We also found that *CTLA-4* haplotypes might influence the development of HCC. In the future, a population-based fine-mapping study with functional assessment should be performed to further verify these correlations, especially for gene-environment and gene-gene interactions.

## Acknowledgments

We appreciate all subjects who participated in this study. We wish to thank Dr Yan Liu (Genesky Biotechnologies Inc, Shanghai, China) for technical support.

## Author contributions

**Conceptualization:** Yuling Sun.

**Data curation:** Chao Liu.

**Methodology:** Yu Chen.

**Project administration:** Jianping Chen.

**Resources:** Jianping Chen.

**Software:** Jiaochun Liu.

**Writing – original draft:** Jing Yang.

**Writing – review and editing:** Weifeng Tang, Jing Yang.

Weifeng Tang orcid: 0000-0002-4157-4057.
